# Glucose and Palmitate Differentially Regulate *PFKFB3*/iPFK2 and Inflammatory Responses in Mouse Intestinal Epithelial Cells

**DOI:** 10.1038/srep28963

**Published:** 2016-07-08

**Authors:** Rachel Botchlett, Honggui Li, Xin Guo, Ting Qi, JiaJia Zhao, Juan Zheng, Shih-Lung Woo, Ya Pei, Mengyang Liu, Xiang Hu, Guang Chen, Ting Guo, Sijun Yang, Qifu Li, Xiaoqiu Xiao, Yuqing Huo, Chaodong Wu

**Affiliations:** 1 Department of Nutrition and Food Science, Texas A&M University, College Station, TX, 77843, USA; 2 Center for Animal Experiment, Wuhan University, Wuhan, Hubei 430071, China; 3 Department of Endocrinology, the First Affiliated Hospital of Chongqing Medical University, Chongqing 400016, China; 4 The Laboratory of Lipid & Glucose Metabolism, the First Affiliated Hospital of Chongqing Medical University, Chongqing 400016, China; 5 Vascular Biology Center, Department of Cellular Biology and Anatomy, Medical College of Georgia, Augusta University, Augusta, GA 30912, USA; 6 Drug Discovery Center, Key Laboratory of Chemical Genomics, Peking University Shenzhen Graduate School, Shenzhen 518055, China

## Abstract

The gene *PFKFB3* encodes for inducible 6-phosphofructo-2-kinase, a glycolysis-regulatory enzyme that protects against diet-induced intestine inflammation. However, it is unclear how nutrient overload regulates *PFKFB3* expression and inflammatory responses in intestinal epithelial cells (IECs). In the present study, primary IECs were isolated from small intestine of C57BL/6J mice fed a low-fat diet (LFD) or high-fat diet (HFD) for 12 weeks. Additionally, CMT-93 cells, a cell line for IECs, were cultured in low glucose (LG, 5.5 mmol/L) or high glucose (HG, 27.5 mmol/L) medium and treated with palmitate (50 μmol/L) or bovine serum albumin (BSA) for 24 hr. These cells were analyzed for *PFKFB3* and inflammatory markers. Compared with LFD, HFD feeding decreased IEC *PFKFB3* expression and increased IEC proinflammatory responses. In CMT-93 cells, HG significantly increased *PFKFB3* expression and proinflammatory responses compared with LG. Interestingly, palmitate decreased *PFKFB3* expression and increased proinflammatory responses compared with BSA, regardless of glucose concentrations. Furthermore, HG significantly increased *PFKFB3* promoter transcription activity compared with LG. Upon *PFKFB3* overexpression, proinflammatory responses in CMT-93 cells were decreased. Taken together, these results indicate that in IECs glucose stimulates *PFKFB3* expression and palmitate contributes to increased proinflammatory responses. Therefore, *PFKFB3* regulates IEC inflammatory status in response to macronutrients.

It is well established that inactivity and overnutrition are major determinants in the development of obesity and contribute to obesity-related metabolic diseases such as type 2 diabetes, fatty liver disease, and inflammatory atherosclerosis^1^,^2^,^3^,^4^,^5^. High saturated fat intake is an especially causative factor as it is known to directly contribute to the growth of individual adipocytes^6^,^7^ which results in impaired lipid storage abilities and the generation of inflammation locally^8^ and systemically in chronic conditions^9^. It is now accepted that the obesity-associated chronic, low-grade systemic inflammation is a major underlying factor for the development of many metabolic diseases^10^,^11^,^12^,^13^,^14^. As such, much research has investigated the mechanisms of diet-induced inflammation, particularly in adipose tissue.

The intestine has recently been implicated as another key organ that critically contributes to the development of obesity-associated chronic inflammation and systemic insulin resistance, and metabolic dysregulation^15^,^16^,^17^,^18^. While investigating how nutrient overload interacts with the intestine to cause systemic inflammation, a number of studies have shown that feeding a high-fat diet (HFD) to mice alters the composition of the gut microbiota and leads to increased intestinal permeability^19^,^20^. This in turn increases the levels of endotoxin in the intestinal lumen and circulation, thereby accelerating obesity and its related metabolic dysregulation. A recent study even indicated a role for HFD-induced intestinal eosinophil depletion, not inflammation, in contributing to defective barrier integrity and the onset of metabolic disease^21^. Considering that the intestine is responsible for digestion, absorption, and assimilation of nutrients and that the nutrients absorbed by intestine have also undergone metabolism whose dysregulation accounts for increased proinflammatory responses, intestinal cells, in particular intestinal epithelial cells (IECs), may respond to nutrient overload to regulate its own inflammatory status prior to regulating inflammatory responses in distal organs. Indeed, in a mouse model of diet-induced obesity (DIO), feeding an HFD activated nuclear factor kappa B (*NFκB*) activity in intestine cells including epithelial cells, immune cells, and endothelial cells of the small intestine^18^. While showing a similar finding in small intestine of HFD-fed mice, the study by Guo *et al*.^22^ further indicated that the anti-inflammatory responses in the intestine accounted for, at least in part, the insulin-sensitizing effect of peroxisome proliferator-activated receptor gamma (*PPARγ*) activation. Given this, there is a need to address the responses of IECs to nutrient overload in order to better understand the mechanisms of obesity-associated inflammation.

In the intestine, the gene 6-phosphofructo-2-kinase/fructose-2, 6-bisphophatase 3 (*PFKFB3*) is abundantly expressed^22^. As the product of *PFKFB3*, inducible 6-phosphofructo-2-kinase (iPFK2) generates fructose-2,6-bisphophate. The latter, as the most powerful activator of 6-phosphofructokinase-1, stimulates glycolysis. Recent studies by Huo *et al*.^13^,^14^,^23^ and Guo *et al*.^13^,^14^,^23^ have further demonstrated that *PFKFB3/*iPFK2 critically determines the balance of metabolic fluxes through glycolysis and fatty acid oxidation, thereby suppressing the generation of reactive oxygen species and proinflammatory responses in adipocytes^13^,^14^,^23^. Unlike its role in adipose tissue/adipocytes, the role for *PFKFB3/*iPFK2 in the small intestine is less known. A previous study by Guo *et al*.^22^ showed increased *PFKFB3* expressions in the small intestine in response to HFD feeding, as well as increased inflammation. However, the regulation of *PFKFB3* within IECs in relation to IEC inflammatory responses has not been investigated. Therefore, this study sought to first determine how macronutrients influence *PFKFB3/*iPFK2 expression in IECs and secondly, how this relates to the IEC inflammatory status.

## Results

### Induction of obesity-related insulin resistance and glucose intolerance

**Figure 1:**
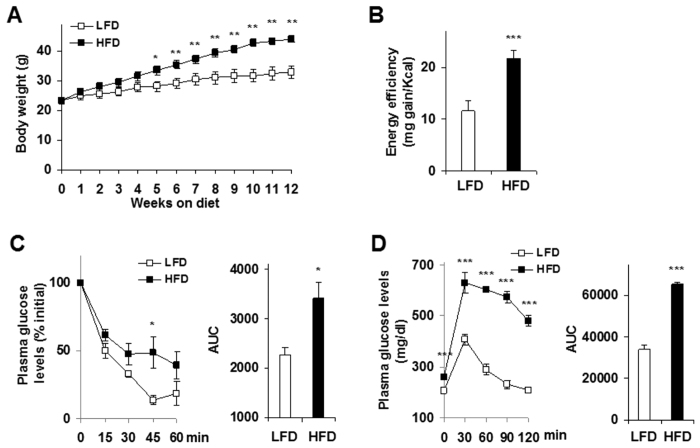
HFD induction of obesity-related insulin resistance and glucose intolerance. Male C57BL/6J mice, at 5–6 weeks of age, were fed an HFD or LFD for 12 weeks, *n *= 9–10. (**A**) Body weight. (**B**) Energy efficiency. (**C**) Insulin tolerance tests. (**D**) Glucose tolerance tests. For (**C,D**), areas under curves (AUC) were calculated based on the corresponding tolerance tests. For (**A–D**), data are means ± SEM. *P < 0.05; **P < 0.01; and ***P < 0.001 HFD vs. LFD (AUC in **C**,**D**) for the same time (**A,C,D**). HFD, high-fat diet; LFD, low-fat diet.

To investigate nutritional regulation of IEC *PFKFB3*/iPFK2 expression and inflammatory responses in the context of obesity and insulin resistance, we fed C57BL/6J mice a HFD for 12 weeks. Compared with low-fat diet (LFD)-fed control mice, HFD-fed mice gained much more body weight (Fig. 1A; *P *< 0.01) after only 5 weeks on the respective diet. During the feeding period, the mice consumed comparable amount of foods, but displayed a significant increase in energy efficiency, which was calculated as milli-gram BW gained/Kcal consumed (Fig. 1B). Along with obesity, HFD-fed mice displayed overt insulin resistance as indicated by the results from insulin tolerance tests (Fig. 1C), in which HFD-fed mice received twice the amount of insulin injection compared with LFD-fed mice. Consistently, HFD-fed mice also showed impairment of glucose tolerance (Fig. 1D). As such, obesity-related insulin resistance and glucose dysregulation were successfully induced in these mice.

### Reduction of *PFKFB3*/iPFK2 in primary IECs

**Figure 2:**
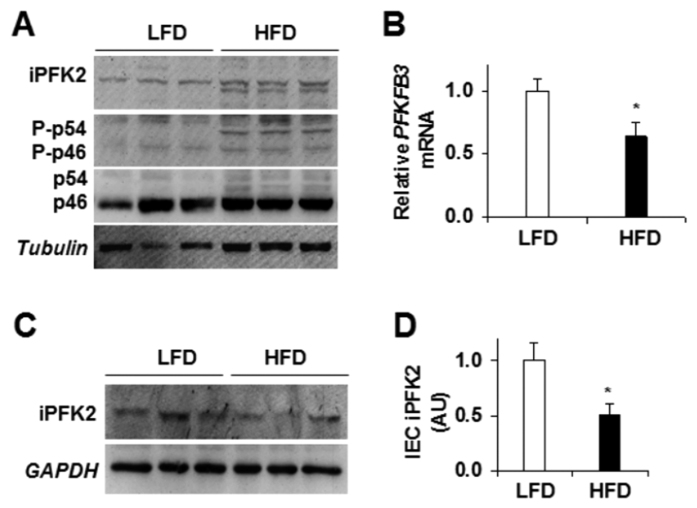
Dietary effects on intestinal *PFKFB3*/iPFK2 expression. Small intestine and primary IECs were isolated following the feeding period. (**A**) Intestine extracts were examined for iPFK2 amount and *JNK* signaling using Western blot analysis. (**B**) IEC expression of *PFKFB3* mRNAs was determined using real-time PCR. (**C**) Western blot analysis of IEC iPFK2 amount. (**D**) Quantification of IEC iPFK2 amount. For bar graphs, data are means ± SEM, *n *= 4–6. HFD, high-fat diet; LFD, low-fat diet; *JNK*, c-Jun n-terminal kinase.

In the present study, we confirmed the previous findings^22^ that HFD feeding increased iPFK2 amount and inflammatory responses in intestine extracts (Fig. 2A). Next, we examined *PFKFB3*/iPFK2 expression in primary IECs isolated from DIO- and/or control mice. Compared with those in IECs from LFD-fed mice, the mRNA levels of *PFKFB3* in IECs from HFD-fed mice were significantly lower (Fig. 2B; *P *< 0.05). Consistently, the amount of iPFK2 was reduced in primary IECs isolated from HFD-fed mice compared with those from LFD-fed mice (Fig. 2C,D). Thus, HFD feeding lowered *PFKFB3*/iPFK2 expression in IECs, which is opposite to the effect of HFD feeding on increasing *PFKFB3*/iPFK2 in intestine extracts^22^. The latter includes various types of cells.

### Stimulation of proinflammatory responses in primary IECs

**Figure 3:**
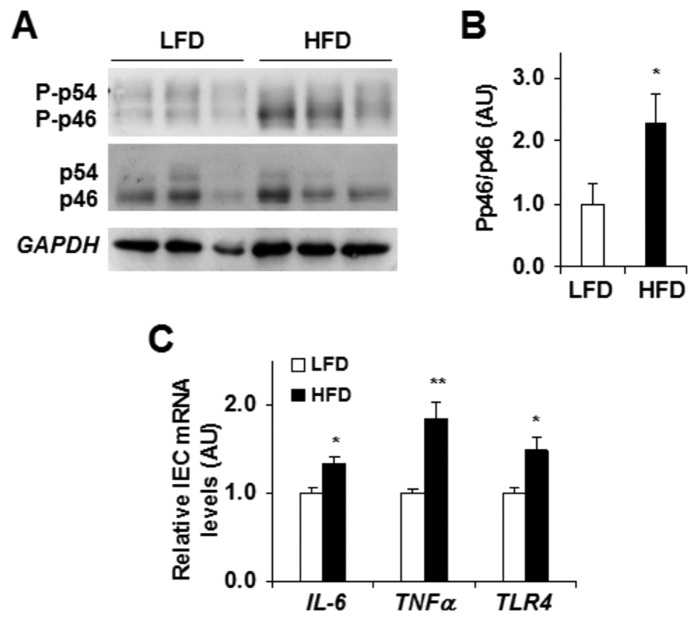
Dietary effects on IEC proinflammatory responses. Primary IECs were isolated following the feeding period. (**A**) Western blot analysis of IEC *JNK* signaling. (**B**) Quantification of IEC Pp46/p46. (**C**) IEC expression of *IL-6*, *TNFα*, and *TLR4* mRNAs. For (**C**) data are means ± SEM, *n *= 4–6. *P < 0.05; **P < 0.01 HFD vs. LFD for the same gene. HFD, high-fat diet; *IL-6*, interleukin-6; iPFK2, inducible 6-phosphofructo-2-kinase; LFD, low-fat diet; Pp46, phosphorylated c-Jun n-terminal kinase 1 (*JNK1*); p46, total *JNK1*; *TLR4*, Toll-like receptor 4; *TNFα*, tumor necrosis factor alpha.

We examined if reduced IEC *PFKFB3*/iPFK2 expression was associated with greater proinflammatory responses. Relative to that of primary IECs of control mice, the proinflammatory signaling through c-Jun N-terminal kinase 1 (*JNK1*) was much higher in primary IECs from DIO mice (Fig. 3A,B). In addition, the mRNA levels of proinflammatory cytokines, e.g., interleukin-6 (*IL*-6) and tumor necrosis factor alpha (*TNFα*), in IECs of DIO mice were significantly higher than in IECs of control mice (Fig. 3C). Similar changes were also observed in the mRNA levels of Toll-like receptor 4 (*TLR4*) (Fig. 3C), whose activation promotes proinflammatory responses. Together, these results suggest that HFD feeding increased IEC proinflammatory responses, which were associated with a reduction in *PFKFB3*/iPFK2 expression in IECs.

### Direct effects of glucose and palmitate on IEC *PFKFB3*/iPFK2 expression and proinflammatory responses

**Figure 4:**
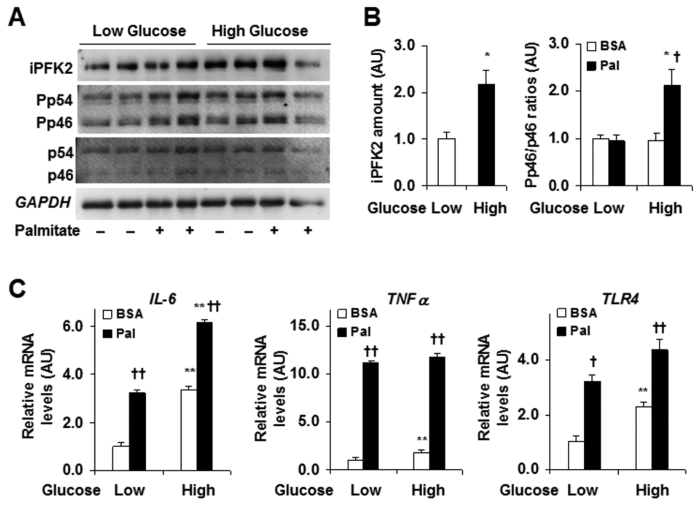
Effects of glucose and palmitate on IEC iPFK2 and proinflammatory responses. CMT-93 cells were treated as described in methods. (**A**) Western blot analyses of IEC iPFK2 amount and *JNK* signaling. (**B**) Quantification of IEC iPFK2 and Pp46/p46. (**C**) IEC expression of *IL-6*, *TNFα*, and *TLR4* mRNAs. For (**B,C**), data are means ± SEM, *n *= 4. *P < 0.05 and **P < 0.01 High glucose vs. Low glucose for the same treatment (BSA or Pal); ^†^*P *< 0.05 and ^††^*P *< 0.01 Pal vs. BSA for the same condition. BSA, bovine serum albumin; HFD, high-fat diet; *IL-6*, interleukin-6; iPFK2, inducible 6-phosphofructo-2-kinase; LFD, low-fat diet; Pal, palmitate; Pp46, phosphorylated c-Jun n-terminal kinase 1 (*JNK1*); p46, total *JNK1*; *TLR4*, Toll-like receptor 4; *TNFα*, tumor necrosis factor alpha.

To gain nutritional insight into *PFKFB3* regulation, we examined the direct effects of glucose and palmitate, two major macronutrients associated with overnutrition, on IEC responses. When the effects of glucose were examined, treatment of CMT-93 cells with 27.5 mmol/L glucose led to greater iPFK2 amount relative to treatment of CMT-93 cells with 5.5 mmol/L glucose, indicating a stimulatory effect of glucose on *PFKFB3* expression (Fig. 4A,B). In contrast, palmitate appeared to mainly account for higher proinflammatory responses in cultured CMT-93 cells. Indeed, two-way ANOVA results indicated an interaction between glucose concentration and palmitate treatment (*P *= 0.010). Specifically, in the presence of low levels of glucose, palmitate did not alter *JNK1* signaling. However, in the presence of high levels of glucose, palmitate treatment caused a significant increase in *JNK1* signaling (Fig. 4A,B). When the expression of proinflammatory cytokines was examined, palmitate treatment led to significantly higher mRNA levels of *TNFα* and *TLR4* regardless of glucose concentration (Fig. 4C). This finding was confirmed by two-way ANOVAs where glucose by treatment interactions were non-significant (*TNFα P *= 0.852; *TLR4 P = *0.847). There was a slight interaction of glucose concentration and palmitate treatment regarding *IL-6* mRNA levels (*P *= 0.048), where mRNA levels were slightly higher in the presence of high concentrations of glucose. Taken together, these results indicate that palmitate, more so than glucose, is responsible for inducing inflammatory responses.

### Stimulatory effect of glucose on *PFKFB3* transcription

**Figure 5:**
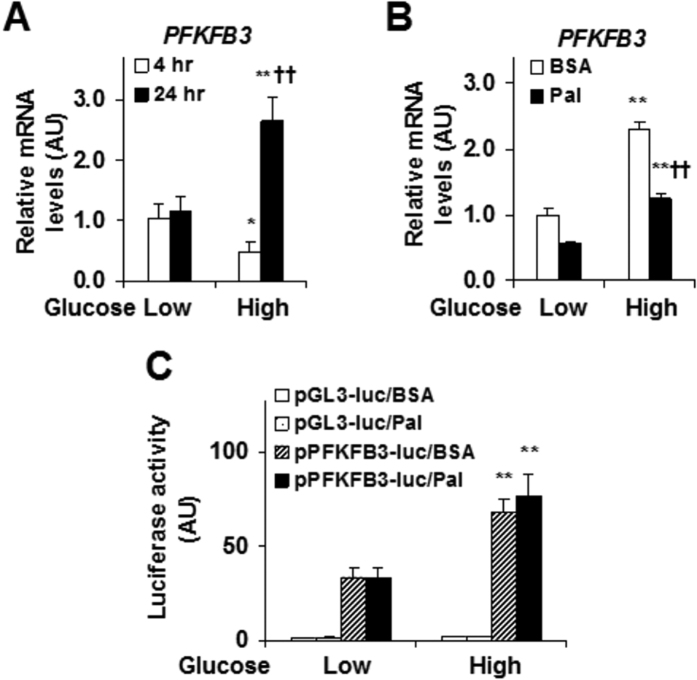
Effects of glucose and palmitate on *PFKFB3* gene transcription. CMT-93 cells were treated as described in the methods. (**A**) Time course and dose responses of glucose regulation of *PFKFB3* expression. (**B**) Effects of glucose and palmitate on *PFKFB3* expression. (**C**) Nutrient regulation of *PFKFB3* promoter activity. For (**A–C**), data are means ± SEM, *n *= 4–6. *P < 0.05 and **P < 0.01 High glucose vs. Low glucose for the same time/treatment (BSA or Pal); ^††^*P *< 0.01 24 hr vs 4 hr (**A**) or Pal vs. BSA (**B**) for the same glucose condition. BSA, bovine serum albumin; Pal, palmitate.

We further explored the effect of glucose on stimulating IEC *PFKFB3* expression. In a time-course study, treatment of CMT-93 cells with low levels of glucose for 24 hr did not alter the mRNA levels of *PFKFB3* compared with treatment of CMT-93 cells with low levels of glucose for 4 hr (Fig. 5A). However, in the presence of high levels of glucose, treatment of CMT-93 cells for 24 hr significantly increased the mRNA levels of *PFKFB3* relative to treatment of CMT-93 cells for 4 hr (Fig. 5A). Next, we examined the effects of palmitate on PFKFB3 expression in the presence of low or high levels of glucose. Two-way ANOVA analyses indicated a significant interaction between glucose concentration and palmitate treatment (*P* = 0.002). Specifically, high levels of glucose remained a dominant effect in stimulating *PFKFB3* expression even in the presence of palmitate, which appeared to decrease the mRNA levels of *PFKFB3* (Fig. 5B). To gain transcription insights, a reporter assay was performed and showed that high levels of glucose stimulated the transcription activity of the *PFKFB3* promoter (Fig. 5C). Unlike glucose, palmitate treatment did not alter the transcription activity of the *PFKFB3* promoter. No interaction between glucose concentration and palmitate treatment was found (*P *= 0.558).

### Suppression of proinflammatory responses by *PFKFB3*/iPFK2 overexpression

**Figure 6:**
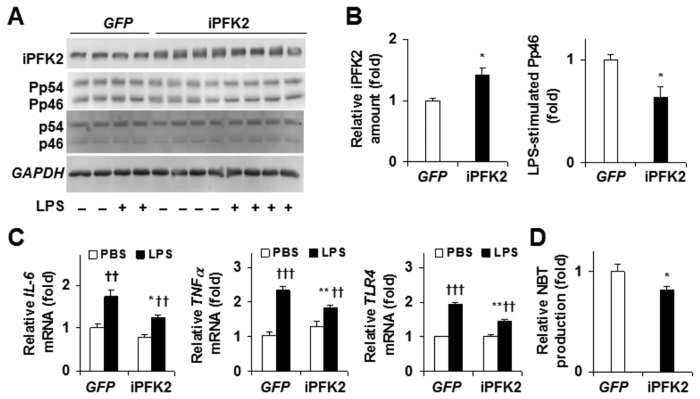
Suppression of proinflammatory responses by *PFKFB3/*iPFK2 overexpression. CMT-93 cells were treated as described in methods. (**A,B**) Western blot analyses for *PFKFB3/*iPFK2 overexpression and quantification of IEC iPFK2 and Pp46/p46. (**C**) Changes in mRNA levels. (**D**) Changes in NBT production. For bar graphs, data are means ± SEM, *n *= 4. *P < 0.05 and **P < 0.01 iPFK2 vs GFP (**B,D**) under the same condition (C, PBS or LPS); ^††^*P *< 0.01 and ^†††^*P *< 0.001 LPS vs PBS for the same treatment (C, *GFP* or iPFK2). For (**A–D**), iPFK2, inducible 6-phosphofructo-2-kinase; Pp46, phosphorylated c-Jun n-terminal kinase 1 (*JNK1*); p46, total *JNK1*; *IL-6*, interleukin-6; *TLR4*, Toll-like receptor 4; *TNFα*, tumor necrosis factor alpha.

We investigated the effect of *PFKFB3*/iPFK2 overexpression on the proinflammatory responses in IECs. Overexpression of *PFKFB3*/iPFK2 was associated with a decrease in *JNK1* signaling (Fig. 6A,B). Also, real-time PCR analyses showed similar results (Fig. 6C). Specifically, LPS-stimulated mRNA levels of all three inflammatory markers in *PFKFB3*-overexpressing cells were significantly lower than those in control cells. *PFKFB3* overexpression also resulted in decreased superoxide production (Fig. 6D), indicated by a decrease in NBT production. Therefore, *PFKFB3* overexpression appeared to decrease the severity of the inflammatory response induced by stress stimuli. Also, it should be pointed out that the relatively low transfection efficiency may lead to underestimation of the effect of iPFK2 overexpression on suppressing IEC proinflammatory responses.

## Discussion

Recent studies have established that *PFKFB3*/iPFK2 links nutrient metabolism and inflammatory responses in several tissues and cell types, e.g., adipocytes and endothelial cells^13^,^14^,^23^,^24^. In the intestine, *PFKFB3*/iPFK2 has also been implicated as a regulator that critically controls the development of intestinal inflammation during obesity^22^. Significantly, *PFKFB3*/iPFK2 is involved in the effect of rosiglitazone, one of the only two currently prescribed medicines as insulin-sensitizers for the treatment of type 2 diabetes, on suppressing intestinal inflammation. The current study presented here builds upon this finding by investigating *PFKFB3*/iPFK2 specifically within IECs, a topic which has not been previously studied.

In DIO mice, the mRNA levels of *PFKFB3* and the amount of iPFK2 were significantly decreased in IECs compared with their respective levels in IECs from LFD-fed mice. Surprisingly, these changes were opposite to the previous finding that the iPFK2 amount was increased in intestine extracts of DIO mice^22^. Considering that the intestine includes various types of cells, it is possible that HFD feeding increased *PFKFB3*/iPFK2 in cells other than IECs, and those cells had higher abundance of *PFKFB3*/iPFK2 than IECs. Additionally, it is possible that during and after the digestion, absorption, and metabolism of nutrients, the composition of nutrients and the metabolites of nutrients were different across IECs and cells other than IECs. As a result, IECs and cells other than IECs likely interacted, respectively, with different nutrients and/or metabolites, thereby displaying differential consequences on *PFKFB3*/iPFK2. While these possibilities need to be further examined, it is important to consider that LFD provides a significantly high amount of carbohydrates (i.e. corn starch). Subsequently, IECs from this diet group would display increased expressions of *PFKFB3*/iPFK2 and thus, exhibit reduced levels in diets with less carbohydrate stimuli (e. g., HFD). This outcome was evident in both *PFKFB3* mRNA levels and iPFK2 amount in primary IECs. As additional evidence, glucose and palmitate showed differential effects on *PFKFB3*/iPFK2 expression in cultured cells (see below). To be noted, the markers of proinflammatory responses, however, were significantly higher with HFD. This effect appeared to be due to the high amount of saturated fat, e.g., palmitate, in the diet, which is known to elevate proinflammatory responses in many tissues/cells^25^,^26^,^27^. In fact, the findings from cultured IEC cell line studies further confirmed this postulation as evidenced by increases in *JNK1* signaling and the mRNA levels of several proinflammatory markers in response to palmitate treatment. Therefore, palmitate/saturated fats appeared to serve as the primary underlying factor as to why IECs displayed an increase in inflammatory responses with HFD feeding. Of importance, the status of proinflammatory responses in IECs was reversely correlated with *PFKFB3*/iPFK2 expression, suggesting that *PFKFB3*/iPFK2 also has an anti-inflammatory role in IECs.

To better understand the underlying mechanisms of dietary effects, we investigated the effects of individual major macronutrients on *PFKFB3*/iPFK2 expression and showed that dietary components exerted differential effects on *PFKFB3*/iPFK2. Notably, glucose, at high levels, significantly increased the mRNA levels of *PFKFB3* and the amount of iPFK2. This stimulatory effect of glucose was expected as *PFKFB3* is highly involved in the stimulation of glycolysis when carbohydrates, in particular glucose, are in excess. In fact, the role of *PFKFB3*/iPFK2 in this manner has been demonstrated in numerous cell types^13^,^24^,^28^. Mechanistically, glucose stimulation of *PFKFB3*/iPFK2 expression was attributable to the effect of glucose on increasing the transcription activity of the *PFKFB3* promoter. In support of this, high levels of glucose significantly increased the activity of luciferase whose expression was driven by a 6.1 kb fragment of the *PFKFB3* promoter. A previous study by Sans *et al*.^29^ had shown that the *PFKFB3* promoter region contains the CACGTG-containing regulatory elements that interact with MondoA:Mlx complex. The latter mediates the effect of glucose on stimulating the expression of a number of genes that participate in glycolysis and lipogenesis when nutrients are in excess. Therefore, within IECs glucose acts to directly stimulate *PFKFB3*/iPFK2 expression. This data further validated the high *PFKFB3*/iPFK2 levels seen in LFD-fed mice.

As a critical component of HFD, palmitate has been previously shown to serve as a ligand to *TLR4* in IECs and therefore directly stimulates the expression of proinflammatory cytokines via the *TGFβ* and *NFκB* pathways^30^. Consistently, we showed that palmitate treatment significantly increased *TLR4* and proinflammatory cytokine expression in cultured IECs. Of interest, palmitate treatment also decreased the mRNA levels of *PFKFB3* and the amount of iPFK2. However, this effect of palmitate was not sufficient to counter against the effect of glucose on increasing *PFKFB3*/iPFK2. Nonetheless, this finding further confirmed that the HFD-induced decrease in *PFKFB3*/iPFK2 expression in primary IECs was due to low levels of carbohydrates (glucose) relative to LFD. In addition, consistent with the results from primary IECs of HFD-fed mice, the decrease in *PFKFB3*/iPFK2 was also correlated with increased proinflammatory responses in cultured IECs. Based on these findings, it is likely that one mechanism for palmitate to increase IEC proinflammatory responses was to decrease *PFKFB3*/iPFK2 expression, thereby leading to a decrease in the anti-inflammatory effect. As a side note, although high glucose (HG) treatment was associated with increased *PFKFB3*/iPFK2, inflammatory markers also remained relatively high at this condition. This was expected as the supra-physiological glucose concentration used (27.5 mmol/L) models overnutrition and was necessary for this study to best demonstrate glucose regulation of *PFKFB3*. However, the control of inflammatory responses induced by such conditions likely requires more than one anti-inflammatory mechanism and thus, the anti-inflammatory ability of *PFKFB3*/iPFK2 alone may not be sufficient to fully suppress an inflammatory response of this nature.

It should be pointed out that the HFD-induced decrease in *PFKFB3*/iPFK2 expression in the primary IECs was reversely correlated with systemic insulin resistance. Based on this relationship, it is likely that *PFKFB3*/iPFK2 in IECs participates in the regulation of obesity-associated insulin resistance and dysregulation of glucose homeostasis. Furthermore, the role played by *PFKFB3*/iPFK2 appears to be attributable to its anti-inflammatory properties as indicated by the results of the present study in both primary IECs and cultured CMT-93 cells, and by the findings of previous studies in adipocytes^13^,^23^. The present study further confirmed this anti-inflammatory role within IECs given that overexpression of *PFKFB3*/iPFK2 decreased proinflammatory markers. Although future studies are needed to validate a specific role for *PFKFB3*/iPFK2 in IECs in the control of obesity-associated insulin resistance and metabolic dysregulation, targeting *PFKFB3*/iPFK2 in IECs through nutritional intervention could offer novel approaches for prevention and/or treatment of inflammatory responses, which contribute to the development of obesity-associated metabolic diseases.

**Figure 7:**
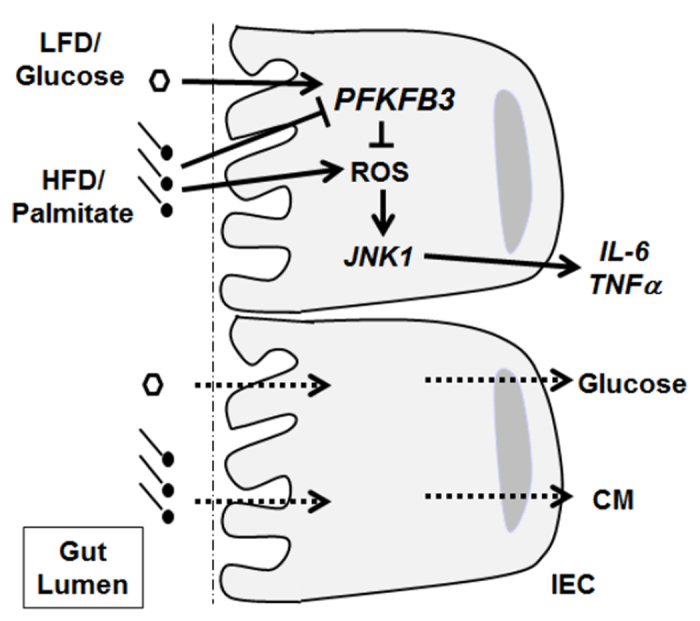
*PFKFB3*/iPFK2 mediates nutritional control of IEC inflammatory responses. The proposed scheme summarizes the differential effects of major macronutrients, e.g., glucose and palmitate, on *PFKFB3*/iPFK2 expression and the inflammatory responses within IECs. Upon LFD feeding, glucose, as a major macronutrient at high concentration, stimulates *PFKFB3*/iPFK2. This in turn inhibits the proinflammatory responses in IECs, likely through suppressing the generation of ROS. Upon HFD feeding, the stimulatory effect on *PFKFB3*/iPFK2 is not present due to low concentrations of glucose. This in turn de-inhibits the proinflammatory responses in IECs. In addition, palmitate, as a major macronutrient of HFD, has a direct proinflammatory effect on IECs. The combined effects exacerbate IEC proinflammatory responses, which may contribute to HFD-induced systemic inflammation. IEC, intestinal epithelial cells; LFD, low-fat diet; HFD, high-fat diet; ROS, reactive oxygen species; *JNK1*, c-Jun N-terminal kinase 1; *IL-6*, interleukin-6; *TNFα*, tumor necrosis factor alpha; *TLR4*, Toll-like receptor 4; CM, chylomicrons.

In summary, this study provides evidence for the first time that major macronutrients, e.g., glucose and palmitate, differentially influence the expression of *PFKFB3*/iPFK2 within IECs. As outlined in the proposed scheme (Fig. 7), glucose directly stimulates *PFKFB3*/iPFK2 expression whereas palmitate more so contributes to the generation of inflammation. The present study also provides the insight into the potential role for *PFKFB3*/iPFK2 in protecting against the proinflammatory responses within IECs. Overall, diet has a significant impact on *PFKFB3*/iPFK2 expression within IECs in the context of obesity-associated inflammation. Because of this, activating *PFKFB3*/iPFK2 in IECs through nutritional approaches could be beneficial for obesity-associated metabolic diseases.

## Materials and Methods

### Animal experiments

C57BL/6J mice were purchased from the Jackson Laboratory (Bar Harbor, ME) and housed under a 12 hr light/dark cycle with free access to water and fed *ad libitum*. At 5–6 weeks of age, male mice were fed either an LFD or HFD for 12 weeks. LFD (Research Diets, New Brunswick, NJ product #D12450B) and HFD (Research Diets, #D12492) consisted of 10% and 60% calories from fat, respectively. The complete macro- and micronutrient composition of the diets is provided in Supplemental Table 1. During the feeding period, body weight and food intake were monitored weekly. After the feeding period, the mice were fasted for 4 hr before sacrifice for collection of blood and tissue samples^31^. Some mice were fasted similarly and used for insulin and glucose tolerance tests and/or IEC isolation as described below. All animals received human care and all study protocols were approved by the Institutional Animal Care and Use Committee of Texas A&M University. In addition, all experiments were performed in accordance with relevant guidelines and regulations.

### Insulin and glucose tolerance tests

Insulin and glucose tolerance tests were performed as previously described^27^,^31^. For insulin tolerance, the mice received an intraperitoneal injection of insulin (0.5 U/kg body weight for LFD-fed mice and 1 U/kg body weight for HFD-fed mice). LFD-fed mice were injected with half the insulin amount as HFD-fed mice since LFD animals are at risk for hypoglycemia following a 1 U/kg dose.

### Isolation of primary IEC

During tissue harvest, the small intestine was removed and cleaned of fecal debris. A small portion of the ileum was prepared for mRNA and protein analyses. The remaining intestine was first flushed with warm DMEM (Sigma-D5523; containing 4 mmol/L L-glutamine, 4.5 g/L glucose, 1.5 g/L sodium bicarbonate, 10% FBS and 1% penicillin/streptomycin), and then placed in warm medium and transferred to a biosafety cabinet. The intestine was cut into 3–4, ~4 cm sections and inverted over bamboo splints. The splints were incubated at 37 °C in DMEM + 2 mmol/L EDTA for 10 min, with gentle shaking every 3 min. After incubation, the splints were discarded, and the medium was filtered through a 70-μm filter and centrifuged at 478 × g for 5 min at 4 °C. After removal of the medium, the IEC pellets were re-suspended in 3 mL warm DMEM and divided into 2, 1.5 mL tubes. After an additional centrifugation, the cell pellets were re-suspended in lysis buffer or STAT-60 for protein and mRNA analyses, respectively.

### Cell culture and treatment

The mouse-derived IEC line, CMT-93 (passage 10–30), was purchased from the American Type Culture Collection (ATCC, Catalog # CRL 223) and grown to confluence in DMEM (Sigma-D5523; containing 4 mmol/L L-glutamine, 4.5 g/L glucose, 1.5 g/L sodium bicarbonate, 10% FBS and 1% penicillin/streptomycin) in 100-mm cell culture dishes in a humidified 5% CO_2_ atmosphere at 37 °C. Confluent cells were transferred to 60-mm cell culture dishes and conditioned in low glucose (LG; 5.5 mmol/L) medium for 24 hr prior to treatment. Thereafter, cells were incubated in LG or high glucose (HG; 27.5 mmol/L) DMEM and treated with palmitate (50 μmol/L) or bovine serum album (BSA, control) for an additional 24 hr. Palmitate was chosen for *in vitro* experiments to mimic the major fat source provided by HFDs. After the treatment period, the cells were harvested for protein and mRNA and stored at −80 °C for further analyses.

To confirm the role of *PFKFB3/*iPFK2 in regulating inflammatory responses, we conducted a gain-of-function *in vitro* experiment. Briefly, cells at 80% confluence were transfected with a plasmid containing the cDNA of iPFK2 with Lipofectamine 2000 transfection reagent (Invitrogen, Carlsbad, CA) following the manufacturer’s protocol. Cells were also transfected with a vector expressing green fluorescent protein (*GFP*) as a control. The transfected cells were then treated with LPS (100 ng/mL) or PBS (control) and harvested and saved in −80 °C for protein and mRNA analyses of proinflammatory markers.

### RNA isolation, reverse transcription, and real-time PCR

Total RNA was isolated from IECs and cultured CMT-93 cells. Reverse transcription was performed using the GoScript™ Reverse Transcription System (Promega) and real-time PCR analysis was performed using SYBR Green (LightCycler^®^ 480 system; Roche Life Science, Indianapolis, IN). The mRNA levels were analyzed for *PFKFB3*, *IL-6*, *TNFα*, and *TLR4*. A total of 0.1 μg RNA was used for the determination. Results were normalized to 18s ribosomal RNA and plotted as relative expression to the mean expression in LG-treated cells or cells of LFD-fed mice, which were set as 1.

### Western blot analysis

Lysates were prepared from frozen IEC samples and cultured cells. Western blot analyses were performed as previously described^32^. Protein amount of iPFK2 (Proteintech Group, Catalog # 13763-1-ap), *JNK1* (p46; Santa Cruz, Catalog # sc-571), and phosphorylated-*JNK1*(p*JNK1*, Pp46; Santa Cruz, Catalog # sc-6254) was examined. The maximum intensity of each band was quantified using ImageJ software. Ratios of Pp46/p46 were normalized to *GAPDH* (Santa Cruz, Catalog # sc-25778) and adjusted relative to the mean of control LFD-fed IEC or LG-treated control cells, which were arbitrarily set as 1 (AU).

### Gene transcription reporter assay

A luciferase reporter assay was performed as previously described^27^. Briefly, a reporter construct in which luciferase expression is driven by an empty promoter (pGL3-luc) or *PFKFB3* promoter (p*PFKFB3*-luc) was transfected into CMT-93 cells. After transfection for 24 h, the cells were incubated with LG or HG medium and treated with palmitate (50 μmol/L) or BSA for an additional 24 hr. Cell lysates were prepared and used to measure luciferase activity using a kit from Promega (Madison, WI). The luciferase activity was normalized to protein concentrations and adjusted relative to the mean of LG- and BSA-treated pGL3 controls, which were arbitrarily set as 1 (AU).

### Nitroblue tetrazolium assay

A nitroblue tetrazolium (NBT) assay was conducted to investigate the role of *PFKFB3*/iPFK2 in regulating superoxide generation. The present study followed a modification of methods previously described^33^.

### Statistical analysis

Numerical data are presented as means ± SEM (standard error). Two-tailed ANOVA or Student’s *t* tests were used to evaluate differences between diet or glucose concentration. To test whether glucose concentration interacted with treatment effects, two-way ANOVAs, and Tukey’s post hoc tests when necessary, were performed. In case of interactions, treatments at individual glucose concentrations were compared. Differences were considered significant at *P *< 0.05.

## Additional Information

**How to cite this article**: Botchlett, R. *et al*. Glucose and Palmitate Differentially Regulate *PFKFB3*/iPFK2 and Inflammatory Responses in Mouse Intestinal Epithelial Cells. *Sci. Rep.* **6**, 28963; doi: 10.1038/srep28963 (2016).

## Supplementary Material

Supplementary Information
