# Supplementary material for: Glucose and Palmitate Differentially Regulate PFKFB3/iPFK2 and Inflammatory Responses in Mouse Intestinal Epithelial Cells
Source: Sci Rep. 2016 Jul 8;6:28963. doi: 10.1038/srep28963 (PMC4937440; doi:10.1038/srep28963)
Supplement: Supplementary Information [file srep28963-s1.pdf]

Glucose and Palmitate Differentially Regulate *PFKFB3*/iPFK2 and Inflammatory Responses in Mouse Intestinal Epithelial Cells

Rachel Botchlett[1], Honggui Li[1], Xin Guo[1], Ting Qi[1], JiaJia Zhao[1], Juan Zheng[1], Shih-Lung Woo[1], Ya Pei[1], Mengyang Liu[1], Xiang Hu[1], Guang Chen[1], Ting Guo[1], Qifu Li[2], Xiaoqiu Xiao[2,3], Yuqing Huo[4,5*], and Chaodong Wu[1*]

[1] Department of Nutrition and Food Science, Texas A&M University, College Station, TX, 77843, USA

[2] Department of Endocrinology and [3] the Laboratory of Lipid & Glucose Metabolism, the First Affiliated Hospital of Chongqing Medical University, Chongqing 400016, China

[4] Vascular Biology Center, Department of Cellular Biology and Anatomy, Medical College of Georgia, Augusta University, Augusta, GA 30912, USA

[5] Drug Discovery Center, Key Laboratory of Chemical Genomics, Peking University Shenzhen Graduate School, Shenzhen 518055, China

Running Title: Intestinal *PFKFB3* and inflammatory responses

Key words: glucose; palmitate; intestinal epithelial cells; *PFKFB3*; obesity; inflammatory response

Correspondence: Chaodong Wu, MD, PhD, College Station, TX 77843; Fax: 979 862 7782; Email: cdwu@tamu.edu; Yuqing Huo, MD, PhD, Augusta, GA 30912; Fax: (706) 721-9799; Email: YHUO@augusta.edu

SUPPLEMENTAL TABLE 1 Macro- and micronutrient composition of low-fat and high-fat diets.

	LFD		HFD	
Macronutrients	g%	kCal%	g%	kCal%
Protein	19.2	20	26	20
Carbohydrates	67.3	70	26	20
Fat	4.3	10	35	60
TOTAL		100		100
kCal/ g	3.85		5.24	
Ingredients				
Casein	200	800	200	800
Corn Starch	315	1260	0	0
Sucrose	350	1400	68.8	275
Cellulose	50	0	50	0
Soybean Oil	25	225	25	225
Lard	20	180	245	2205
Mineral Mix S10026	10	0	10	0
Vitamin MixV10001	10	40	10	40
